# Hospitals with 150 Beds and Upwards

**Published:** 1918-12-21

**Authors:** 


### HOSPITAL NEEDS AND HOSPITAL APPEALS.

### Hospitals with 150 Beds and Upwards.

**CITY OF LONDON HOSPITAL FOR DISEASES OF THE CHEST, VICTORIA PARK, E.**—The pressing character of the various structural improvements and additions, which unfortunately had to be curtailed on the outbreak of war, comprising the completion of an additional block, containing surgical beds and a new Operation Theatre, the improvement of the sanitary arrangements in one wing of the Hospital, and the substitution of electric for gas lighting throughout the building, makes it imperative that the earliest possible effort towards their completion should be made. Additional facilities for research work are also in contemplation. The hospital has deserved well of the public by reason of its valuable co-operation in respect of needs arising out of the war. A large number of men, principally those invalided from the Forces by reason of having broken down with tuberculosis, have been treated, besides the services of a number of members of the staff being given in the Army and Navy Medical Services. The heavy increase in the cost of maintaining the hospital has unfortunately absorbed a balance of income which it had been hoped to apply to the building additions and renovations. The Committee has therefore to appeal for a sum estimated at about £15,000 in order to carry out the improvements. The Treasurer, Sir G. Wyatt Truscott, Bt., will gladly receive contributions towards the Maintenance Fund, the needs for which have increased by over £5,000 per annum, and also the Special Fund for the building improvements. Bankers: Barclays Bank, Ltd., 54 Lombard Street, E.C. 3.

**GREAT NORTHERN CENTRAL HOSPITAL, HOLLOWAY ROAD, N. 7.**—The committee of management has had under consideration for some time an extension which will be a war memorial for the district. This step is essential if the hospital is to cope with the many and increasing claims upon it from the densely populated districts of North London. Even when wounded soldiers are no longer patients, the hospital cannot provide adequately for the needs of the immense area it serves, and now more must be done for the ever-increasing number of discharged disabled soldiers and sailors in North London, the treatment of whom the committee have undertaken. The scheme of extension has been submitted to the King Edward's Hospital Fund for London, who have approved of the issue of an appeal. Secretary: Mr. Gilbert G. Panter.

**GUY'S HOSPITAL, S.E. 1.**—In-patients to the number of 8,892 and 97,267 out-patients were relieved by this famous institution in 1917, at a cost of £105,354 and with an assured income of about £54,000. Owing to the increased cost of maintenance and a largely diminished income from legacies, there was a deficiency on the year of nearly £13,000. The Governors will, therefore, greatly welcome any help at this season towards preventing in 1918 such a deficit as might impair the full service of the hospital. President, Mr. H. Cosmo O. Bonsor; treasurer, Viscount Goschen; bankers, the Bank of England.

**KING'S COLLEGE HOSPITAL, LONDON, S.E. 5.**—During the four years of war 29,000 wounded officers and men and 12,000 civilian in-patients have been nursed; 114,000 civilian out-patients have been treated. With regard to the future, the wards now occupied by the military will gradually be transferred to the civil side, and it is hoped to greatly extend the work that has been kept up for civilian patients. In addition, arrangements are being made for giving massage and electrical treatment to Chelsea Pensioners, and soldiers returned to civil life, while special attention will be devoted to the children of the neighbourhood, for whom the Swedish remedial department is doing wonders. The Committee make an earnest appeal for £100,000 to pay off the debt on the new buildings, and at least £20,000 extra annually to provide for current expenses, as its present assured income is very small. £5,000 is urgently required before the end of the year to enable the hospital to meet its immediate oligations. Donations should be forwarded to the Chairman, King's College Hospital, Denmark Hill, S.E. 5.

**LONDON HOMŒOPATHIC HOSPITAL.**—The board of management make a special appeal for aid in making good the large deficits incurred during the past few years. Eighty-five beds have been placed at the disposal of the Admiralty for sick and wounded sailors, of whom some 1,600 have already been treated to date. The receipt of contributions, addressed to the Earl of Donoughmore, the Treasurer, or Mr. Edward A. Attwood, the Secretary, at the hospital, Great Ormond Street, W.C. 1, will be gratefully acknowledged.

**NATIONAL HOSPITAL FOR THE PARALYSED AND EPILEPTIC.**—The annual expenditure of this hospital has risen from £18,000 to close upon £30,000, mainly in consequence of the increase in the prices of food and drugs. Seventy soldiers suffering from shell shock, blindness, mutism, deafness, nerve injuries, and general nervous complaints are to be found in the wards. The beds have been increased to 170 to meet the extra call for in-patient treatment. Three smaller hospitals at Maidenhead, Clapham Park, and in Bloomsbury for discharged disabled soldiers are being managed for the Ministry of Pensions, and there is a country branch for civilian patients at East Finchley. A sum of £10,000 is required to meet obligations to the bank and to tradesmen. The Secretary is Mr. Godfrey H. Hamilton, Queen Square, W.C.

**ROYAL FREE HOSPITAL, GRAY'S INN ROAD, W.C.**—The committee are making strenuous efforts to reduce the debt to the hospital bankers, at present amounting to £20,500, and they urgently need support for maintenance, and for extension of the maternity and infant welfare work. Mr. Reginald R. Garratt, Secretary.

**ST. BARTHOLOMEW'S HOSPITAL, WEST SMITHFIELD, LONDON, E.C. 1.**—Is in need of further support. The maintenance expenses have steadily increased since the commencement of the war, and it is estimated that the total expenditure of the hospital and convalescent home for the current year will be not less than £112,000, as compared with an average annual expenditure of £85,000 during the three years immediately preceding the war. Treasurer, the Viscount Sandhurst, P.C.; bankers, the Bank of England.

**ST. GEORGE'S HOSPITAL, HYDE PARK CORNER, S.W. 1.**—The committee earnestly appeals for contributions, preferably annual subscriptions, for the general maintenance of the institution. The enormous increase in

**Hospital Needs and Hospital Appeals**—(*continued*).

the cost of all things necessary for the efficient treatment of the patients is likely to continue for some considerable time longer, and, as the income does not show a corresponding increase, the energetic help of all who have the interests of the hospital at heart is absolutely essential in order that its good work for the sick poor may not be impaired. Secretary : Mr. James M. Churchfield.

**ST. MARY'S HOSPITAL, PADDINGTON, W.**—The regular sources of income yield about £18,000 only. A further sum of £22,000, therefore, is required every year. While this hospital was doing a great work for the war, its annual subscribers fell off through the war to the extent of more than £700 a year. Its war work has consisted not only in the devotion of one-third of its beds to the treatment of wounded soldiers from the Front, but in the manufacture and supply of anti-typhoid and other vaccines to all the Allied Armies in the field. Millions of doses have been supplied since the commencement of the war. To this work was due, in great measure, the striking rarity of typhoid fever in the Allied Forces. Concurrently with this work for the men in the field, the hospital is providing, and continues to provide, for large numbers of discharged disabled men both in the wards and in the out-patient department. Secretary, Mr. Thomas Ryan.

**SEAMEN'S HOSPITAL, GREENWICH, S.E., AND ALBERT DOCKS.**—395 sailors are under treatment in these hospitals. About half the number are Naval ratings from the Fleet, the other half being the equally gallant men of the Merchant Service, to whose courage and contempt of danger we all owe so much. Our obligation to the mariner in the future will be as great as before. Not only have we to look to him to clear the seas of mines but also to renew our trade and carry the merchandise which alone can bring prosperity again to this country. Secretary, Mr. P. Michelli, C.M.G.

**WESTMINSTER HOSPITAL, BROAD SANCTUARY, S.W.**—Since the beginning of the year 398 military patients have been received, and owing to the great advance in the price of supplies an appeal is made for generous support. Secretary, Mr. S. M. Quennell.

